# Dynamics of antibodies to SARS‐CoV‐2 in convalescent plasma donors

**DOI:** 10.1002/cti2.1285

**Published:** 2021-05-16

**Authors:** Maurice Steenhuis, Gerard van Mierlo, Ninotska IL Derksen, Pleuni Ooijevaar‐de Heer, Simone Kruithof, Floris L Loeff, Lea C Berkhout, Federica Linty, Chantal Reusken, Johan Reimerink, Boris Hogema, Hans Zaaijer, Leo van de Watering, Francis Swaneveld, Marit J van Gils, Berend Jan Bosch, S Marieke van Ham, Anja ten Brinke, Gestur Vidarsson, Ellen C van der Schoot, Theo Rispens

**Affiliations:** ^1^ Department of Immunopathology Sanquin Research Amsterdam The Netherlands; ^2^ Landsteiner Laboratory Amsterdam University Medical Centre University of Amsterdam Amsterdam The Netherlands; ^3^ Department of Experimental Immunohematology Sanquin Research and Landsteiner Laboratory Amsterdam University Medical Centre Amsterdam The Netherlands; ^4^ Department of Infectious Diseases Public Health Service region Utrecht Utrecht The Netherlands; ^5^ Department of Virology Sanquin Diagnostic Services Amsterdam The Netherlands; ^6^ Sanquin Blood Supply Foundation and Amsterdam University Medical Centre Amsterdam The Netherlands; ^7^ Sanquin Blood Bank Unit Transfusion Medicine Leiden The Netherlands; ^8^ Department of Transfusion Medicine Sanquin Blood Bank Amsterdam The Netherlands; ^9^ Department of Medical Microbiology Amsterdam UMC University of Amsterdam Amsterdam The Netherlands; ^10^ Virology Division Department of Infectious Diseases and Immunology Faculty of Veterinary Medicine Utrecht University Utrecht The Netherlands

**Keywords:** ACE2‐competitive ELISA, antibodies, COVID‐19, longitudinal, neutralisation

## Abstract

**Objectives:**

Characterisation of the human antibody response to SARS‐CoV‐2 infection is vital for serosurveillance purposes and for treatment options such as transfusion with convalescent plasma or immunoglobulin products derived from convalescent plasma. In this study, we longitudinally and quantitatively analysed antibody responses in RT‐PCR‐positive SARS‐CoV‐2 convalescent adults during the first 250 days after onset of symptoms.

**Methods:**

We measured antibody responses to the receptor‐binding domain (RBD) of the SARS‐CoV‐2 spike protein and the nucleocapsid protein in 844 longitudinal samples from 151 RT‐PCR‐positive SARS‐CoV‐2 convalescent adults. With a median of 5 (range 2–18) samples per individual, this allowed quantitative analysis of individual longitudinal antibody profiles. Kinetic profiles were analysed by mixed‐effects modelling.

**Results:**

All donors were seropositive at the first sampling moment, and only one donor seroreverted during follow‐up analysis. Anti‐RBD IgG and anti‐nucleocapsid IgG levels declined with median half‐lives of 62 and 59 days, respectively, 2–5 months after symptom onset, and several‐fold variation in half‐lives of individuals was observed. The rate of decline of antibody levels diminished during extended follow‐up, which points towards long‐term immunological memory. The magnitude of the anti‐RBD IgG response correlated well with neutralisation capacity measured in a classic plaque reduction assay and in an in‐house developed competitive assay.

**Conclusion:**

The result of this study gives valuable insight into the long‐term longitudinal response of antibodies to SARS‐CoV‐2.

## Graphical abstract

By measuring IgG concentrations at many time points per individual (844 samples for 151 subjects), we showed not only that the decline in IgG antibodies to SARS‐CoV‐2 occurs at substantially different rates between individuals, but also that rates of decline of antibody levels diminish as time progresses beyond 6 months, indicative of long‐term memory.

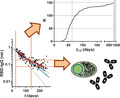

## Introduction

Severe acute respiratory syndrome coronavirus 2 (SARS‐CoV‐2) is the causative agent of the ongoing coronavirus disease (COVID‐19) pandemic emerged in Wuhan (China) in December 2019. SARS‐CoV‐2 is classified under the *Betacoronavirus* 2B and is closely related to SARS‐CoV (> 80% genomic similarity) and MERS‐CoV (50% genomic similarity), which have caused previous outbreaks.^1^, ^2^ COVID‐19 is associated with a wide spectrum of disease severity, ranging from asymptomatic to acute respiratory distress syndrome, and is already responsible for more than 1 million deaths worldwide.^3^

Besides vaccination, prevention of infection with SARS‐CoV‐2 might be achieved by transfusion with plasma collected from individuals after recovery from COVID‐19 (COVID‐19 convalescent plasma, CCP) or immunoglobulin products derived from CCP.^4^, ^5^, ^6^ This therapy would be especially relevant for immunocompromised individuals. CCP therapy is safe,^7^ and it has been approved by the FDA for treatment. The clinical effect especially in severely ill patients seems to be limited.^8^ The most potent CCP units are in theory those containing the highest amounts of neutralising antibodies against SARS‐CoV‐2. To select convalescent plasma donors with high neutralising antibody titres, it is important to understand the dynamics of antibodies against SARS‐CoV‐2 in the period after recovery from SARS‐CoV‐2.

Virtually all PCR‐confirmed patients develop IgM, IgA and IgG antibodies against the virally encoded surface glycoproteins spike (S) and nucleocapsid protein (NP).^9^, ^10^, ^11^ The S protein mediates binding of the virus particle to angiotensin converting enzyme‐2 (ACE2) on target cells through its receptor‐binding domain (RBD),^12^, ^13^ facilitating viral entry. A large fraction of anti‐S antibodies is directed against RBD, of which many are neutralising.^14^, ^15^ Anti‐S antibodies binding outside of the RBD may also contribute to neutralisation.^14^, ^15^ In addition, antibody levels seem to vary depending on the infection duration and severity of disease.^16^

Seroprevalence studies have demonstrated that antibodies to SARS‐CoV‐2 can be detected up to at least 3–8 months after disease recovery.^17^, ^18^, ^19^, ^20^ However, seroprevalence depends on the characteristics of the study population, and many studies do not report on the quantitative aspects of the antibody response. Several short‐term studies consistently show a seroconversion of IgG, IgA and IgM antibodies against the viral proteins S and NP within 1–3 weeks after symptom onset, depending on disease severity.^9^, ^10^, ^11^, ^21^ Less detailed information is available about the long‐term course of antibody titres. Several studies investigated the longitudinal antibody response and found that 1 month after onset of symptoms, antibody levels reach a plateau followed by rapidly declining IgM and IgA titres, whereas IgG titres seem to remain high up to 6 months.^18^, ^19^, ^22^, ^23^, ^24^, ^25^, ^26^ A recent study by Dan *et al*.^20^ in which patients were followed up to 8 months consistently showed only a modest decline in anti‐S IgG titres and neutralising antibody titres. Limitations of these studies include a, sometimes, small number of subjects, and in particular the low number of longitudinal data points available for each subject, which restricts the possibilities to analyse trends in antibody levels over time.

Here, we collected samples from 151 RT‐PCR‐positive SARS‐CoV‐2‐recovered adults donating convalescent plasma over a study period of up to 34 weeks. The median number of samples per donor was 5 (IQR 4–7; range 2–18), which allowed a more detailed and quantitative analysis of individual trends over time.

## Results

In this study, a total of 151 adult CCP donors were included to examine the longitudinal antibody response to SARS‐CoV‐2. All subjects were tested RT‐PCR‐positive (nasopharyngeal swab) for SARS‐CoV‐2 (baseline information can be found in Table 1). On average, the first donation was collected 59 days after onset of COVID‐19 symptoms. Additional plasma samples per donor were sequentially collected for up to 250 days after onset of symptoms, resulting in 844 plasma samples in total. Of these, 20 samples were excluded as outliers (for details, see Methods), resulting in 676 samples for *period 1* (up to 157 days after symptom onset; last sample median 110 days after symptom onset) and another 148 samples for *period 2* (extending to 250 days after symptom onset; Supplementary figure 1). During period 2, many donors were no longer donating because of insufficient titres, and this extended range is thus only available for a biased selection of plasma donors (*n* = 55). Analysis of the antibody prevalence to SARS‐CoV‐2 was therefore focused on period 1.

**Table 1:** Baseline table for the convalescent plasma donors used in this study

	Total (*n* = 151)	Male (*n* = 105)	Female (*n* = 46)	*P*	Non‐Hosp. (*n* = 128)	Hosp. (*n* = 23)	*P*
Age (years)	45 (35–55)^a^	48 (36–55)	42 (29–51)	0.058	42 (31–53)	54 (48–58)	< 0.001
Male (%)	70				64	100	< 0.001
Non‐hosp. (%)	85	78	100	< 0.001			
Median first donation (days after symptom onset)	59 (47–74)	59 (48–74)	54 (38–80)	0.61	54 (45–74)	63 (52–76)	0.13
IgG‐RBD (AU mL^−1^)^b^	24 (9–70)	30 (10–89)	17 (9–28)	0.009	18 (8–39)	113 (70–223)	< 0.001
IgG‐NP (AU mL^−1^)^b^	28 (8–92)	35 (10–105)	16 (6–56)	0.012	19 (7–66)	117 (45–300)	< 0.001
IgM‐RBD (%)^b^	49	55	35	0.024	45	70	0.027
IgA‐RBD (%)^b^	65	70	55	0.075	60	91	0.004
Half‐life RBD IgG (days)^c^	62 (48–87)	59 (47–84)	69 (53–99)	0.027	64 (49–93)	54 (47–68)	0.053
Half‐life NP IgG (days)^c^	59 (44–82)	58 (43–78)	67 (50–108)	0.023	60 (45–84)	54 (42–64)	0.12

^a^ Numbers between parentheses indicate interquartile range.

^b^ Value at first donation; antibody titre in AU mL^−1^ for IgG‐RBD and IgG‐NP, seroprevalence for IgM‐RBD and IgA‐RBD.

^c^ Values obtained from regression analysis.

### Seroprevalence of antibodies against RBD

**Figure 1:**
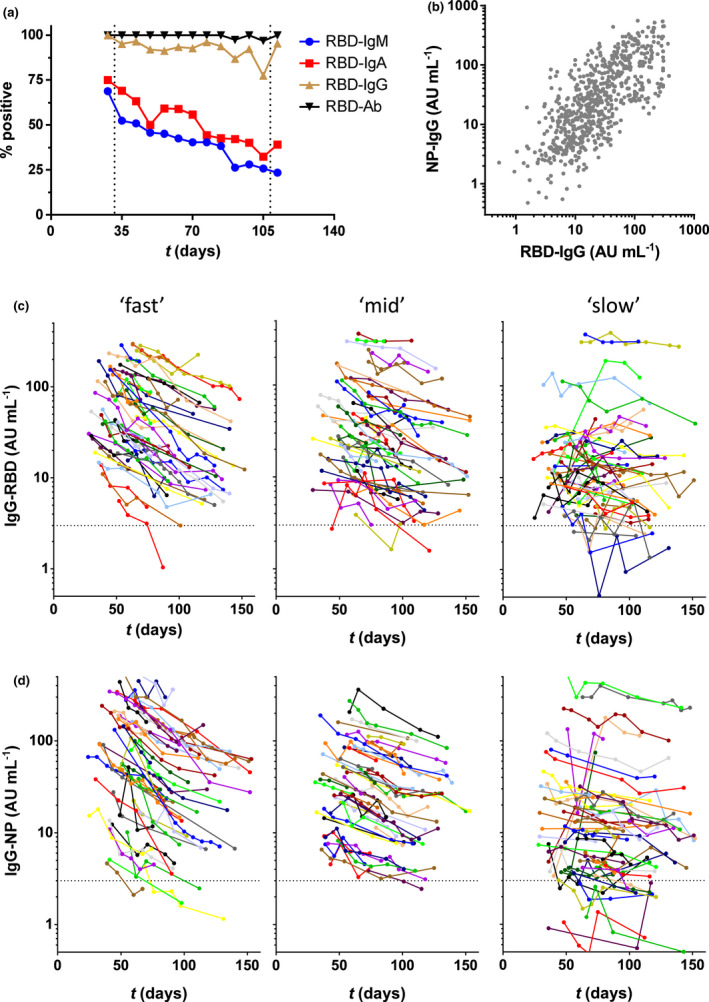
Antibodies against SARS‐CoV‐2 in CCP donors during up to 157 days of follow‐up (period 1). **(a)** Seropositivity was assessed by isotype‐specific (IgM, IgA and IgG) assays and the total antibody assay RBD‐Ab. Results were stratified per week postonset symptoms; samples covering < 5 and > 15 weeks (< 32 and > 109 days) were combined. **(b)** Correlation between anti‐RBD and anti‐NP IgG levels. Samples from the CCP donors were tested in the RBD and NP IgG isotype‐specific assay, and their correlation was evaluated by Spearman's rank test (*r* = 0.73, *P* < 0.001). **(c)** Concentrations of IgG anti‐RBD and **(d)** anti‐NP plotted in days after onset of disease symptoms (676 samples from 151 donors). Left, middle and right panels contain samples that were stratified according to fitted half‐lives of antibody levels (see Figure 2a and b for explanation of ‘slow’, ‘mid’ and ‘fast’). All data represent the mean of at least 2 independent replicates.

To study seroprevalence to SARS‐CoV‐2, we used our recently described sensitive total antibody bridging assay (so‐called RBD‐Ab) to RBD.^9^ All donors were positive for SARS‐CoV‐2 antibodies as assessed with the RBD‐Ab assay, except for two samples, which belong to one donor that seroreverted at week 13 (day 90) after a positive test at week 10 (day 70) postsymptom onset (Figure 1a; Supplementary table 1). In addition, using isotype‐specific assays^9^ we determined seroprevalence of IgA, IgG and IgM to RBD. All samples collected before week 5 were seropositive for IgG against RBD (Figure 1a). As time progressed, less than 10% of samples fell below the detection limit for IgG up to week 13, indicative that IgG levels remain in circulation for substantial periods after recovery.^18^, ^19^ In contrast, the amount of samples that were seropositive in the IgA and IgM isotype‐specific assay was 75% and 68.8% before week 5, respectively, and gradually further declined during the study period, in line with previous studies.^18^, ^19^

### Levels of IgG antibody responses against RBD and NP

To obtain better insight into the antibody response, we quantitatively analysed the SARS‐CoV‐2 IgG antibodies against NP and RBD in all 151 adult donors. For (relative) quantification, we made use of pooled plasma of CCP donors as calibrator. As shown in Figure 1b, antibody concentrations varied > 100‐fold between individuals for both anti‐RBD and anti‐NP and were significantly correlated. High concentrations of antibody were significantly associated with hospitalisation (Table 1).^27^, ^28^ Furthermore, antibody levels were significantly higher in men than in women. However, this is largely because of the fact that only men were hospitalised, and no significant difference was observed between non‐hospitalised men and women (22 vs 17 AU mL^−1^ and 22 vs 16 AU mL^−1^ for anti‐RBD and anti‐NP, respectively; *P* = 0.28 and 0.14).

We also measured the IgG1 and IgG3 subclass responses to RBD at first donation in subclass‐specific ELISAs. To properly allow quantitative comparison, we recombinantly expressed IgG1 and IgG3 monoclonal antibodies,^15^ which were then used as a calibrator. We observed that IgG anti‐RBD consisted mainly of IgG1, with only a median of 2.5% (IQR 1.5–5.1%; Supplementary figure 3a) for IgG3. Since higher subclass ratios were reported for antibodies against the S2 vs S1 domain of the S protein,^29^, ^30^ we also tested IgG1 and IgG3 in a subset of samples against the full S protein, but we obtained very similar results (Supplementary figure 3b).

Since titrations of the recombinant antibodies were parallel to the plasma pool used as calibrator (Supplementary figure 3d), this also enabled us to estimate the absolute concentration of RBD‐reactive IgG antibodies. The average concentration of anti‐RBD IgG observed in CP donors of 24 AU mL^−1^ was found to reflect approximately 2–3 µg mL^−1^.

### Dynamic changes in IgG against SARS‐CoV‐2

**Figure 2:**
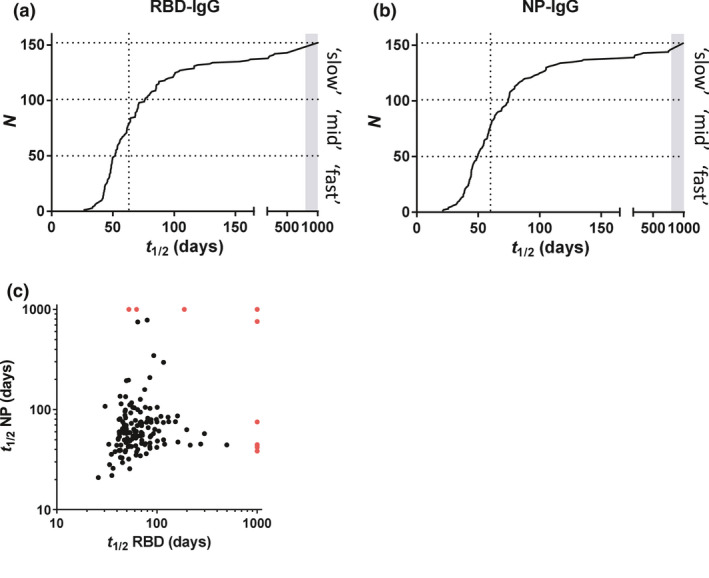
Regression analysis of IgG levels. For both **(a)** RBD IgG and **(b)** NP IgG, data of period 1 were modelled using a mixed‐effects model (log‐linear in IgG; random intercept and slope, time as fixed variable). Slopes of IgG decay in time (see Figure 1) were converted into half‐lives. Dotted vertical lines indicate median half‐lives. Boundaries between ‘slow’, ‘mid’ and ‘fast’ (used to stratify data in Figure 1) are 50 and 76 days, and 48 and 73 days for RBD and NP, respectively. **(c)** The correlation between estimated half‐lives for anti‐RBD IgG and anti‐NP IgG levels was evaluated by Spearman's rank test (*r* = 0.23, *P* = 0.0037). In case of rising levels, *t* was arbitrarily assigned a value of 1000 in the above images (indicated by the grey bar **(a**, **b)** and red dots **(c)**, respectively).

Next, we assessed the dynamics of the IgG antibody response over time. Within the time window of up to 157 days (period 1), most individuals demonstrated a steady and approximately log‐linear decline in antibody concentration (Figure 1c and d). Assuming a log‐linear (i.e. first‐order) decline in antibody concentrations, we analysed this trend using linear mixed‐effects modelling, which enabled us to calculate individual half‐lives. Half‐lives were found to vary several‐fold between individuals, with a median half‐life of 62 (IQR 48–87) and 59 (IQR 44–82) days for anti‐RBD and anti‐NP IgG levels, respectively (Figure 2a and b and Table 1). There is a very weak correlation between estimated half‐life for anti‐RBD IgG and anti‐NP IgG levels, as shown in Figure 2c (Spearman's *r* = 0.23, *P* = 0.0037). However, the subset of individuals demonstrating the most rapid decline does so for both types of antibodies. In addition, there is a significant but weak negative correlation between half‐life and absolute levels (Supplementary figure 2), indicating a more rapid decline in individuals with the highest antibody levels.

Although IgG3 has a shorter half‐life than IgG1, we did not observe an association between IgG clearance rate and IgG1 and IgG3 levels in the first sample (Supplementary figure 3c), also not for those samples with a relatively high percentage of IgG3 antibodies. In fact, the half‐lives of the IgG anti‐RBD and anti‐NP levels exceed the intrinsic half‐life of both IgG3 antibodies (c. 7 days) and IgG1 antibodies (c. 21 days),^31^ in line with continued antibody production.

**Figure 3:**
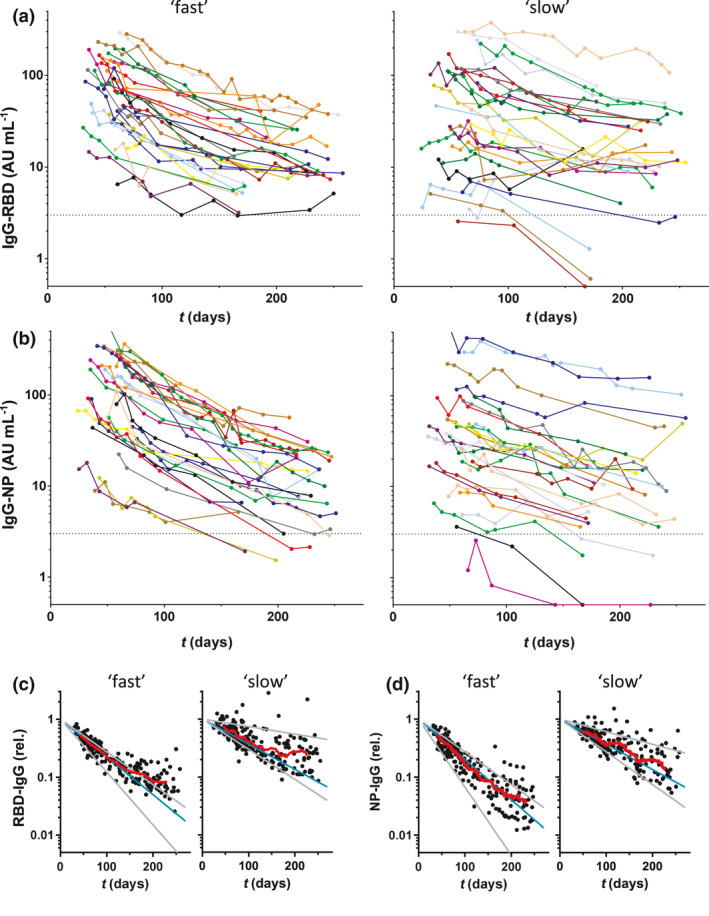
Dynamic changes in IgG antibodies against SARS‐CoV‐2 during extended follow‐up up to 250 days (period 2). Concentrations of **(a)** IgG anti‐RBD and **(b)** anti‐NP plotted in days after onset of disease symptoms (430 samples from 55 donors; mean of 2 independent replicates). Left panels, 28 donors with the fastest decline during period 1, and right panels, the slowest 27 donors, and the boundary between ‘fast’ and ‘slow’ was 56 and 55 days for RBD and NP, respectively. **(c**, **d)** Same data but normalised per donor using fitted intercepts from regression analysis of period 1. Blue and grey lines indicate median, smallest and largest fitted slopes from the same analysis (excluding positive slopes, 2 for RBD and 1 for NP) within both groups. Red lines are running averages showing an overall trend within both groups of donors.

For a subset of 55 donors, extended follow‐up was available for up to 250 days (period 2). Of note, as explained in the Methods, there is a selection bias in this group towards higher antibody levels for period 2. Overall, the declining trends in antibody levels continued (Figure 3a and b). Interestingly, the rate of decline appears to decrease at later time points, especially for donors with an initially high decline (Supplementary figure 4). The effect is more pronounced for anti‐RBD (Figure 3c) than for anti‐NP (Figure 3d).

### IgG levels correlate with viral neutralisation

**Figure 4:**
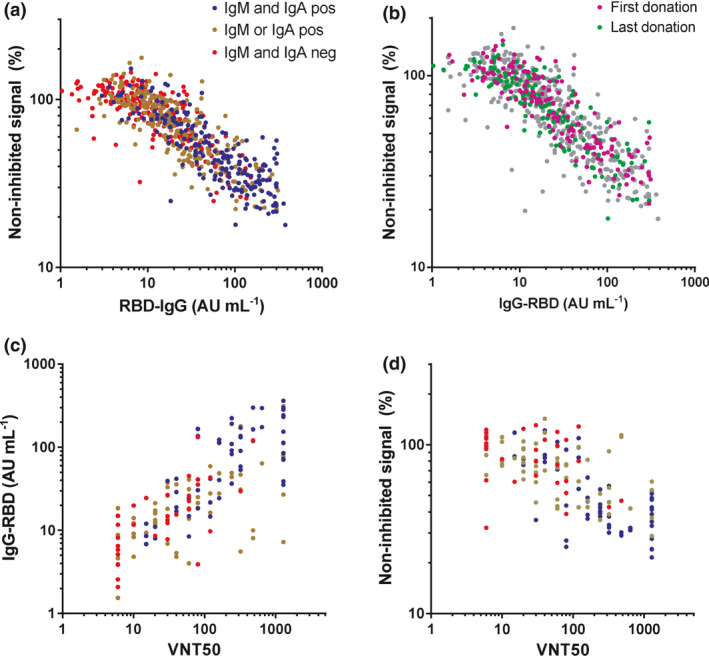
Correlation between IgG levels and virus neutralisation. Plasma samples were tested in the in‐house developed competitive ELISA (676 samples from 151 individual donors) and in the classic plaque reduction assay (147 samples from 129 individual donors; mean of 2 independent replicates). The correlation between anti‐RBD IgG and virus neutralisation in the **(a**, **b)** competitive assay and **(c)** plaque reduction assay was assessed by Spearman's rank test (*r* = 0.85, *r* = 0.75, respectively, *P* < 0.001). **(d)** A correlation between the two viral neutralisation assays was also observed (Spearman's *r* = 0.65, *P* < 0.001).

To obtain insight into the neutralising capacity of the antibody response in this population, we carried out a competitive ELISA in which binding of RBD to ACE2, the receptor on SARS‐CoV‐2 target cells, is inhibited by blocking antibodies derived from plasma samples (Supplementary figure 5). We observed a high and significant correlation between anti‐RBD IgG levels and the amount of competition (Figure 4a), independent of the timing of plasma collection (Figure 4b). Samples negative for both IgA and IgM showed less inhibition than samples positive for both. However, correlation between anti‐RBD IgM or IgA levels and the strength of competition was very weak (Supplementary figure 6). This indicates that anti‐RBD IgG probably plays a dominant role in neutralising SARS‐CoV‐2 virus particles, especially in the long term, because of low or absent levels of anti‐RBD IgM and IgA. Furthermore, we tested virus neutralisation of a subset of the plasma samples (*n* = 147) using the well‐established classic plaque reduction assay using live SARS‐CoV‐2 virus. We observed a good correlation between plaque reduction, expressed as the titre that reduced plaque formation by 50% (VNT50), with anti‐RBD IgG (Figure 4c) and with inhibition of receptor binding (Figure 4d) in the in‐house developed competitive assay. An IgG level above 19 AU mL^−1^ provides *c*. 100% (78–100%) sensitivity for detectable neutralisation (with specificity 50–58%). These data also indicate that our ELISA‐based competitive assay could be used to evaluate the virus‐neutralising capacity of anti‐SARS‐CoV‐2 antibodies in plasma samples.

## Discussion

Here, we describe the individual dynamics of the longitudinal antibody response in plasma samples from CCP donors that were followed for up to 250 days postonset of COVID‐19 disease symptoms. IgG levels against RBD and NP decreased only gradually, albeit with substantial interindividual variation, and the decrease appeared to slow down as time progressed. This information not only is relevant in the context of the persistence of detectable antibody responses over time and the assessment of seroprevalence and immunity in populations, but may also be used to improve the selection procedure of CCP donors for therapies with convalescent plasma. Furthermore, insight into the variable antibody dynamics may also benefit the evaluation of vaccine responses.

The median half‐lives of 62 (48–87) and 59 (44–82) days for anti‐RBD and anti‐NP IgG, respectively, are reasonably similar if slightly shorter to those found in Dan *et al*.^20^ that reported a half‐life of 83 days (62–127) for anti‐RBD IgG and 67 days (49–105) for anti‐NP IgG. The difference may be explained by the fact that Dan *et al*. only used paired samples from individuals who donated 6 days and 8 months postonset of symptoms. Our study included a median number of five samples per donor, which allowed a more thorough analysis of individual rate profiles. The data suggest that the decline in antibody levels is not uniform during this 8‐month period, but continues at a slower pace at later time points. This finding indicates that next to the presence of a pool of short‐lived antibody‐secreting cells that vanishes over time, a pool of long‐lived plasma cells is formed. Interestingly, individuals showed a considerable, several‐fold difference in IgG half‐lives, possibly because of intersubject variation in the relative contribution of short‐lived antibody‐secreting cells to the overall antibody production. A limitation of this study is the substantial uncertainty in the individual half‐life estimates, since the available data will sometimes only cover a fraction of a half‐life.

Long‐term maintenance of antibody production is mainly provided by a pool of long‐lived plasma cells that can last a lifetime.^32^ In general, several time scales of declining antibody concentrations may be identified, of which the longest lasting can have half‐lives in the order of many years and thus beyond the scope of the current study.^33^ Nevertheless, as alluded to above, our results hint at induction of long‐lasting antibody production following SARS‐CoV‐2 infection. It will be interesting to continue following the decline in anti‐RBD IgG in time and investigate whether the longevity of the antibody response is similar to other coronaviruses, such as SARS‐CoV that can still be detected in most individuals 3 years after recovery.^34^ The antibody titre required for protection against reinfection in humans is not yet known and has to be evaluated also in the context of a recall response upon reinfection. Recent studies provide evidence that upon infection, individuals develop SARS‐CoV‐2‐specific memory B and memory T cells that can be detected for up to 240 days, with numbers of IgG memory B cells increasing over time and plateauing after *c*. 150 days.^20^, ^35^ Taken together, the relationship between long‐lasting antibody production and the T‐ and B‐cell memory compartments requires more investigation.

We also quantitatively examined the IgG1 and IgG3 subclass response and found that in our study population, IgG1 appeared to be by far the dominant IgG subtype. This is in contrast to previous studies that suggest an IgG subclass ratio skewed towards IgG3 during SARS‐CoV‐2 infection.^30^, ^36^ A possible explanation for this discrepancy is that in these studies, the plasma samples were collected shortly after COVID‐19 disease recovery, while in our study, samples were collected up to 157 days after onset of disease symptoms. In addition, a strength of the current study is the use of IgG1 and IgG3 monoclonal anti‐S antibodies with identical variable domains, which allowed reliable quantification of the relative amounts of IgG1 and IgG3.

The dynamics of the SARS‐CoV‐2 antibodies play an important role in CCP donor recruitment in order to select the most optimal timing of plasma collection. Potent CCP units should in theory contain high amounts of neutralising antibodies against SARS‐CoV‐2. We observed a good correlation between anti‐RBD IgG and both a classic plaque reduction viral neutralisation assay, consistent with previous studies,^37^, ^38^ and a straightforward competitive ELISA that indirectly assesses viral neutralisation by measuring the ability of plasma containing anti‐SARS‐CoV‐2 antibodies to prevent interaction between RBD and ACE2. This suggests that a competitive assay may also reliably report on viral neutralisation. Of note, a recent study by Gasser *et al*.^39^ found that depletion of IgM resulted in a substantial loss of virus neutralisation of the corresponding plasma samples, suggesting a role of IgM in virus neutralisation. However, IgM (and IgA) levels will drop relatively fast after recovery. Indeed, many plasma donations did not contain detectable amounts of IgM or IgA. In line with this, we observed only a very weak correlation between viral neutralisation in the competitive assay and IgM or IgA levels.

In line with other studies, higher IgG levels were found for patients that were hospitalised than those with milder symptoms not requiring hospitalisation, indicating that patients recovered from severe illness are more suitable for CCP donation.^21^ Nevertheless, there are potential caveats for these donors. First, a recent study by Larsen *et al*. showed that severely ill COVID‐19 patients with acute respiratory distress syndrome may display a so‐called ‘afucosylated IgG anti‐S response’.^40^, ^41^ In addition, multiple studies reported the presence of autoantibodies against a.o. type I IFN‐α2 and IFN‐ω in patients with severe COVID‐19 pneumonia who also have high IgG anti‐SARS‐CoV‐2 titres.^42^, ^43^, ^44^, ^45^, ^46^ These parameters might negatively impact the outcome of therapies with convalescent plasma and suggest that it may be safer to rely on convalescent plasmas from patients with mild symptoms, despite the fact that those tend to have lower antibody response.

In conclusion, this study provides insight into the individual dynamics of antibody levels to SARS‐CoV‐2 during up to 250 days after symptom onset. Substantial (several‐fold) variation in individual half‐lives was observed, with median half‐lives of about 60 days for both anti‐RBD and anti‐NP IgG, and a tendency towards slower rates of decline of antibody levels as time progressed.

## Methods

### Samples and donors

Plasma samples were obtained from RT‐PCR‐positive SARS‐CoV‐2‐recovered adult individuals (*n* = 151) donating convalescent plasma at Sanquin Blood Bank (Amsterdam, the Netherlands) who enrolled the CCP programme of Sanquin between 30 March 2020 and 6 September 2020. Donors were included if tested positive in a SARS‐CoV‐2 PCR, which during this period was only provided to individuals presenting COVID‐19‐related symptoms, and were symptom‐free for at least 2 weeks. Details are provided in Table 1. Inclusion was contingent upon the availability of at least two samples with minimally 30 days in between, and samples were collected between 30 March and 14 August 2020 (period 1), yielding 694 samples. Additional samples were collected based on availability for 55 donors up to 11 November 2020 (period 2), yielding another 150 samples. During this period, many donors dropped out because of insufficient titres. Some donors with low titres continued as a regular plasma donor. The median number of samples per donor was 5 (IQR 4–7; range 2–18).

Data and samples were collected only from voluntary, non‐remunerated, adult donors as described previously^47^ and who provided written informed consent as part of routine donor selection and blood collection procedures, which were approved by the Ethics Advisory Council of Sanquin Blood Supply Foundation. This study has been conducted in accordance with the ethical principles set out in the declaration of Helsinki, and all participants provided written informed consent.

### Total and isotype‐specific antibody ELISA

Total antibody, IgM and IgA to RBD were measured as described previously.^9^ S, RBD and NP proteins were produced as described before,^9^, ^40^ IgG to S, RBD and NP were measured essentially as described before,^9^ but with the following modifications. IgG1 and IgG3 subclasses were detected by adding 0.5 µg mL^−1^ HRP‐conjugated monoclonal mouse antihuman IgG1 (MH161.1, Sanquin, Amsterdam) or 1.0 µg mL^−1^ antihuman IgG3 (MH163.1, Sanquin, Amsterdam) diluted in PBS supplemented with 0.02% polysorbate‐20 and 0.3% gelatine) (PTG) for 30 min. Samples were tested with 1:1200 dilution for IgG to RBD and NP, and IgG1 to RBD, and 1:100 dilution for IgG3 to RBD. A cut‐off was based on the 98 percentile of signals of 240 pre‐outbreak plasma samples. Signals were quantified using a serially diluted calibrator consisting of pooled convalescent plasma that was included on each plate. This calibrator was arbitrarily assigned a value of 100 AU mL^−1^. For anti‐RBD IgG1 and IgG3, the calibrator consisted of recombinantly expressed IgG1 and IgG3 monoclonal antibody. Both antibodies had the same variable regions (clone COVA1‐18), but were engineered with different heavy chains.^15^, ^48^ Results were expressed as arbitrary units (AU) per mL (AU mL^−1^) and represent a semi‐quantitative measure of the concentrations of IgG antibodies.

### Competitive ELISA

Neutralising capacity of SARS‐CoV‐2 antibodies was assessed using a competitive assay. Serum samples were incubated at 125‐fold dilution for 60 min with 5 ng mL^−1^ biotinylated RBD in PTG. Next, 100 μL aliquots were transferred to MaxiSORP microtitre plates (Thermo Fisher Scientific, Waltham, MA, USA) coated with 300 ng mL^−1^ ACE2 and incubated for 60 min. The ACE2 was produced in HEK cells with a HAVT20 leader peptide, 10xhis‐tag and a BirA‐tag as described by Dekkers *et al*.^48^. After washing five times with PBS supplemented with 0.02% polysorbate‐20 (PBS‐T), plates were incubated for 30 min with streptavidin‐poly‐HRP (M2032, Sanquin). Plates were washed five times with PBS‐T, and 100 μL of TMB substrate (100 μg mL^−1^) and 0.003% (v/v) hydrogen peroxide (Merck, Darmstadt, Germany) in 0.11 m sodium acetate buffer (pH 5.5) were added to each well. A total of 100 μL of 0.2 m H_2_SO_4_ (Merck) was added to stop the reaction. Absorbance was measured at 450 and 540 nm. The difference was used to evaluate RBD binding. As such, results were corrected for background signals (absence of RBD) and expressed as percentage binding relative to the uninhibited condition (% non‐inhibited signal).

### Neutralisation assay

Sera were tested by a SARS‐CoV‐2‐specific virus neutralisation test (VNT) based on a protocol described previously with some modifications.^49^ In brief, replicate serial dilutions of heat‐inactivated samples (30 min at 56°C) were incubated with 100‐fold tissue culture 50% infectious dose of SARS‐CoV‐2 for 1 h at 35°C. African green monkey (Vero‐E6) cells were added in a concentration of 2 × 10^4^ cells per well and incubated for 3 days at 35°C in an incubator with 5% carbon dioxide. The 50% virus neutralisation titre (VNT50), defined as the highest serum dilution that protected more than 50% of cells from cytopathological (lysed cells) effect, was taken as the neutralisation titre. Samples with titres ≥10 were defined as SARS‐CoV‐2 seropositive.

### Data processing and analysis

Initial evaluation of IgG levels resulted in identification of 20 samples for which we observed either a > 4‐fold drop in level/week or 4‐fold difference/2 week with consecutive follow‐up before and after. Since such drastic changes in IgG level are unlikely to represent basal trends in IgG production, these were excluded from all further analyses.

For the remaining 676 samples collected between 30 March and 14 August, the longitudinal changes in IgG antibody levels were analysed by linear mixed‐effects modelling in R (v3.6.0; www.r‐project.org) using the LmerTest package (v3.1.2). We assumed first‐order decay in concentrations and therefore a linear trend in the log‐transformed concentrations with time. Time was used as fixed variable, with random intercept for subject and random slope for time to account for individual clearance rates.

Statistical analyses were carried out using GraphPad Prism 7 (Graphpad software, San Diego, CA, USA). Subgroup analysis on individual baseline parameters (Table 1) was performed using either a Mann‐Whitney *U‐*test or *Z‐*test for proportions. Correlations were evaluated as the Spearman rank‐order coefficient.

## Conflict of interest

The authors declare no conflict of interest.

## Author contributions

**Maurice Steenhuis:** Investigation; Visualization; Writing‐original draft; Writing‐review & editing. **Gerard van Mierlo:** Investigation; Writing‐review & editing. **Ninotska Derksen:** Investigation; Writing‐review & editing. **Pleuni Ooijevaar de Heer:** Investigation; Writing‐review & editing. **Simone Kruithof:** Investigation; Writing‐review & editing. **Floris Loeff:** Methodology; Supervision; Writing‐review & editing. **Lea C Berkhout:** Data curation; Project administration; Writing‐review & editing. **Federica Linty:** Investigation; Writing‐review & editing. **Chantal Reusken:** Supervision; Writing‐review & editing. **Johan Reimerink:** Supervision; Writing‐review & editing. **Boris Hogema:** Data curation; Supervision; Writing‐review & editing. **Hans Zaaijer:** Supervision; Writing‐review & editing. **Leo van de Watering:** Data curation; Writing‐review & editing. **Francis Swaneveld:** Writing‐review & editing. **Marit van Gils:** Supervision; Writing‐review & editing. **Berend Jan Bosch:** Supervision; Writing‐review & editing. **S Marieke van Ham:** Conceptualization; Supervision; Writing‐review & editing. **Anja ten Brinke:** Conceptualization; Supervision; Writing‐review & editing. **Gestur Vidarsson:** Conceptualization; Supervision; Writing‐review & editing. **C Ellen van der Schoot:** Conceptualization; Supervision; Writing‐review & editing. **Theo Rispens:** Conceptualization; Supervision; Visualization; Writing‐review & editing.

## Data Availability

For original data, please contact the Corresponding author.
