# Supplementary material for: Dynamics of antibodies to SARS‐CoV‐2 in convalescent plasma donors
Source: Clin Transl Immunology. 2021 May 16;10(5):e1285. doi: 10.1002/cti2.1285 (PMC8126762; doi:10.1002/cti2.1285)
Supplement: Supplementary file 1 [file CTI2-10-e1285-s001.docx]

# Supporting information

# Dynamics of antibodies to SARS-CoV-2 in convalescent plasma donors

**Authors**

Maurice Steenhuis^1^, Gerard van Mierlo^1^, Ninotska IL Derksen^1^, Pleuni Ooijevaar-de Heer^1^, Simone Kruithof^1^, Floris L Loeff^1^, Lea C Berkhout^1^, Federica Linty^2^, Chantal Reusken^5^, Johan Reimerink^5^, Boris Hogema^9^, Hans Zaaijer^4^, Leo van de Watering^7^, Francis Swaneveld^3^, Marit J van Gils^6^, Berend Jan Bosch^8^, Marieke van Ham^1^, Anja ten Brinke^1^, Gestur Vidarsson^2^, Ellen C van der Schoot^2^, Theo Rispens^1, *^

**Affiliations**

^1^Department of Immunopathology, Sanquin Research, Amsterdam, The Netherlands, and Landsteiner Laboratory, Amsterdam University Medical Centre, University of Amsterdam, Amsterdam, The Netherlands.

^2^Department of Experimental Immunohematology, Sanquin Research and Landsteiner Laboratory Amsterdam University Medical Centre, 1066 CX Amsterdam, the Netherlands.

^3^Department of Transfusion Medicine, Sanquin Blood Bank, 1066 CX Amsterdam, the Netherlands.

^4^Sanquin Blood Supply Foundation and Amsterdam University Medical Centre, Amsterdam, the Netherlands.

^5^Department of Infectious Diseases, Public Health Service region Utrecht, Utrecht, the Netherlands.

^6^Department of Medical Microbiology, Amsterdam UMC, University of Amsterdam, Amsterdam, the Netherlands.

^7^Sanquin Blood Bank, Unit Transfusion Medicine, Leiden, the Netherlands.

^8^Virology Division, Department of Infectious Diseases and Immunology, Faculty of Veterinary Medicine, Utrecht University, 3584 CL Utrecht, the Netherlands.

^9^Department of Virology, Sanquin Diagnostic Services, Amsterdam, The Netherlands.

**Corresponding author**

Theo Rispens; Department of Immunopathology, Sanquin Research, Amsterdam, The Netherlands, and Landsteiner Laboratory, Amsterdam University Medical Centre, University of Amsterdam, Amsterdam, The Netherlands; T.Rispens@sanquin.nl.

**Supplementary figure 1. Sample collection in period 1 and 2.**

**Supplementary figure 2. Correlation plots.**

Plots for fitted intercepts and slopes for (**a, b**) RBD-IgG and (**c, d**) NP-IgG with respect to measured IgG levels at first donation. Interaction between slope and intercept/baseline IgG level: *r* = 0.22, *P* = 0.006 (RBD) and *r* = 0.34, *P* < 0.0001 (NP).

**Supplementary figure 3. Contribution of IgG1 and IgG3 subclasses to anti-RBD.**

IgG1 and IgG3 antibody subtypes were measured against (**a**) RBD and (**b**) S1 and displayed as ratio IgG3 compared to IgG1. For RBD, first available sample for each donor was measured, for S, a random selection of 30 donors was selected. (**c**) IgG3/IgG1 ratio vs clearance rate. (**d**) Titrations of IgG1 and IgG3 versions of the monoclonal antibody 1-18, and the plasma pool (PP) that was used as calibrator in this study.

**Supplementary figure 4. Normalized individual trends in IgG antibody levels during extended follow-up up to 250 days (period 2).**

Concentrations of (**a**) IgG anti-RBD and (**b**) anti-NP, normalized per donor using fitted intercepts from regression analysis (Figure 2), plotted in days after onset of disease symptoms (430 samples from 55 donors). Left panels 28 donors with fastest decline during first 20 weeks, right panels slowest 27 donors.

**Supplementary figure 5. Overview of the competition assay**.

(**a**) schematic representation of the assay format. Biotinylated RBD will bind to immobilized ACE2 only to the extent that neutralizing antibodies from serum do not inhibit this interaction. (**b**) dose-response curves for several sera containing antibodies to Sars-CoV-2.

**Supplementary figure 6. Correlations of IgM and IgA anti-RBD with IgG anti-RBD and ACE2-RBD competition.**

Correlation between IgM (**a**) and IgA (**b**) with IgG anti-RBD in 676 samples of 151 donors collected up to week 20 after symptom onset. Spearman *r* = 0.41 (*P* < 0.0001), and 0.23 (*P* < 0.001), resp. Correlation between competition ELISA and IgM (**c**) and IgA (**d**) in the same set of samples. Spearman *r* = -0.26 (*P* < 0.001), *r* = 0.22 (*P* < 0.001), resp.

**Supplementary table 1.** Seroprevalence of anti-RBD antibodies

|  | wk | **<5** | **5** | **6** | **7** | **8** | **9** | **10** | **11** | **12** | **13** | **14** | **15** | **>15** |
| --- | --- | --- | --- | --- | --- | --- | --- | --- | --- | --- | --- | --- | --- | --- |
| IgG | N | 47 | 42 | 59 | 75 | 69 | 76 | 55 | 51 | 48 | 38 | 26 | 31 | 64 |
|  | % Pos | 100 | 95 | 97 | 92 | 91 | 93 | 93 | 96 | 94 | 87 | 92 | 77 | 95 |
| IgA | N | 48 | 42 | 57 | 70 | 71 | 73 | 52 | 52 | 47 | 38 | 25 | 31 | 64 |
|  | % Pos | 75 | 69 | 63 | 50 | 59 | 59 | 56 | 44 | 43 | 42 | 40 | 32 | 39 |
| IgM | N | 48 | 42 | 57 | 70 | 71 | 73 | 52 | 52 | 47 | 38 | 25 | 31 | 64 |
|  | % Pos | 69 | 52 | 51 | 46 | 45 | 42 | 40 | 40 | 38 | 26 | 28 | 26 | 23 |
| Ab | N | 47 | 42 | 59 | 75 | 69 | 75 | 55 | 51 | 48 | 38 | 26 | 31 | 64 |
|  | % Pos | 100 | 100 | 100 | 100 | 100 | 100 | 100 | 100 | 100 | 97 | 100 | 97 | 100 |
